# Supplementary material for: A multiscale 3D chemotaxis assay reveals bacterial navigation mechanisms
Source: Commun Biol. 2021 Jun 3;4:669. doi: 10.1038/s42003-021-02190-2 (PMC8175578; doi:10.1038/s42003-021-02190-2)
Supplement: Supplementary file 1 — Supplementary Information [file 42003_2021_2190_MOESM1_ESM.pdf]

Supplementary Information

"A multiscale 3D chemotaxis assay reveals bacterial navigation mechanisms"

Marianne Grognot & Katja M. Taute

Supplementary Figure 1

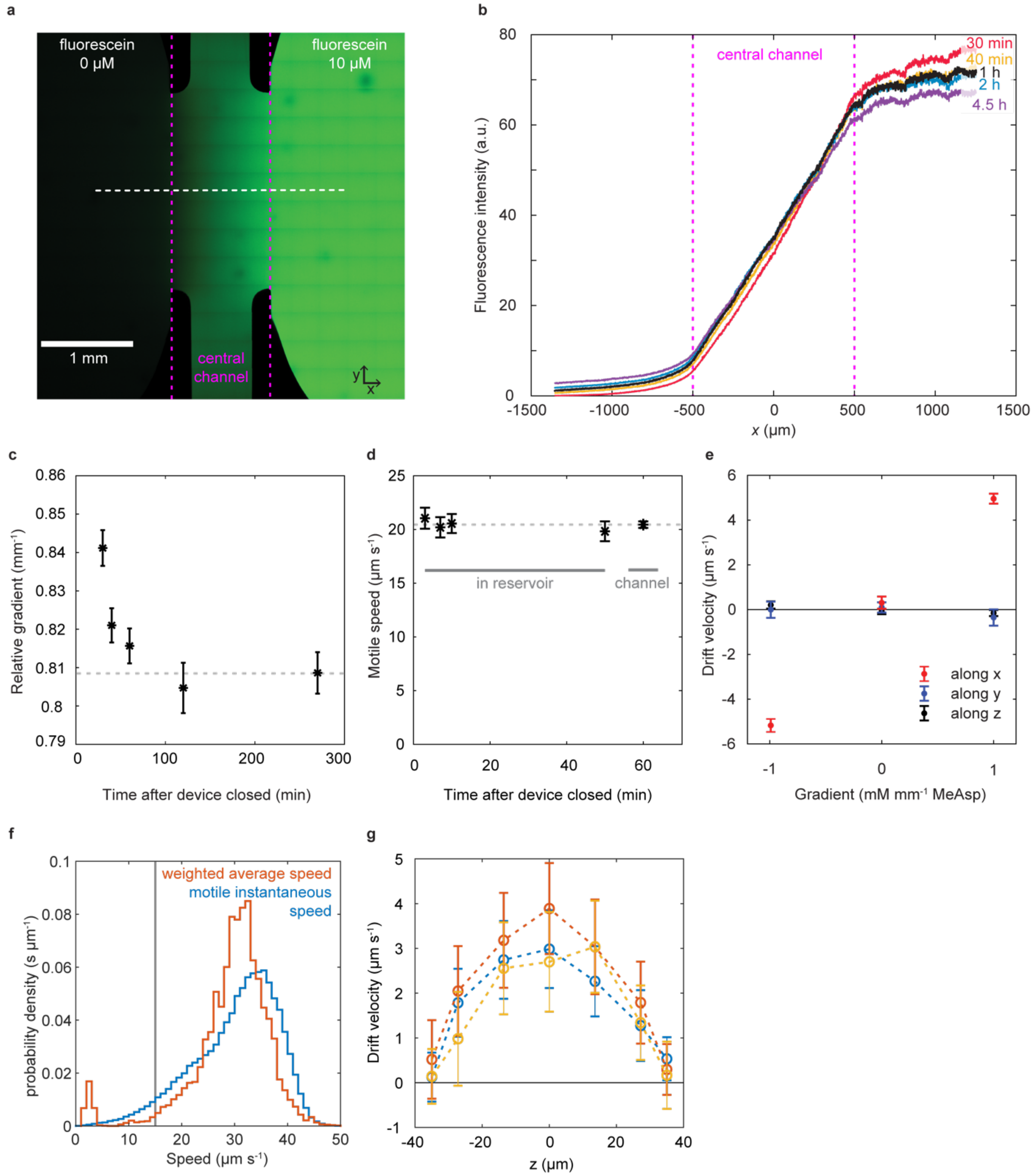

Supplementary Figure 1: Characterization and validation of chemotaxis assay. a) Top view of chemotaxis chamber with fluorescein gradient, stitched from tiled confocal fluorescence images. The gradient is established in a 1 mm long central channel (magenta) between two large reservoirs (left and right). White dashed line indicates approximate line scan shown in panel b. b) Temporal establishment and stability of the fluorescein gradient assessed by confocal

fluorescence imaging along a line in the center of the channel. c) Relative gradient in the center of the device as a function of time after closing the device, normalized relative to the intensity in the right reservoir. d) Mean swimming speed of the *E. coli* motile population (defined as having a mean swimming speed larger than 10 µm s^{-1}) over time after closing the device. Gray dashed lines in panels c and d serve to guide the eye. e) Average *E. coli* bacterial velocities measured in all 3 dimensions in the presence and absence of a 1 mM/mm MeAsp gradient, and when the device is rotated by 180° about the *z* axis. The magnitude of the measured drift up the gradient changes by less than 1% when the chamber is flipped. All error bars are standard errors of the mean. f) Distribution of individual average swimming speeds for the full population of bulk trajectories (red, weighted by trajectory duration) and of instantaneous swimming speeds for the motile population (blue), defined as having an average speed larger than a threshold (grey), set at 15 µm s^{-1} for this experiment. Data shown are for the same experiment as for Fig. 1b. g) *E. coli* drift velocity versus *z* position is reproducible between the three replicates combined in Fig. 1d.

Supplementary Figure 2

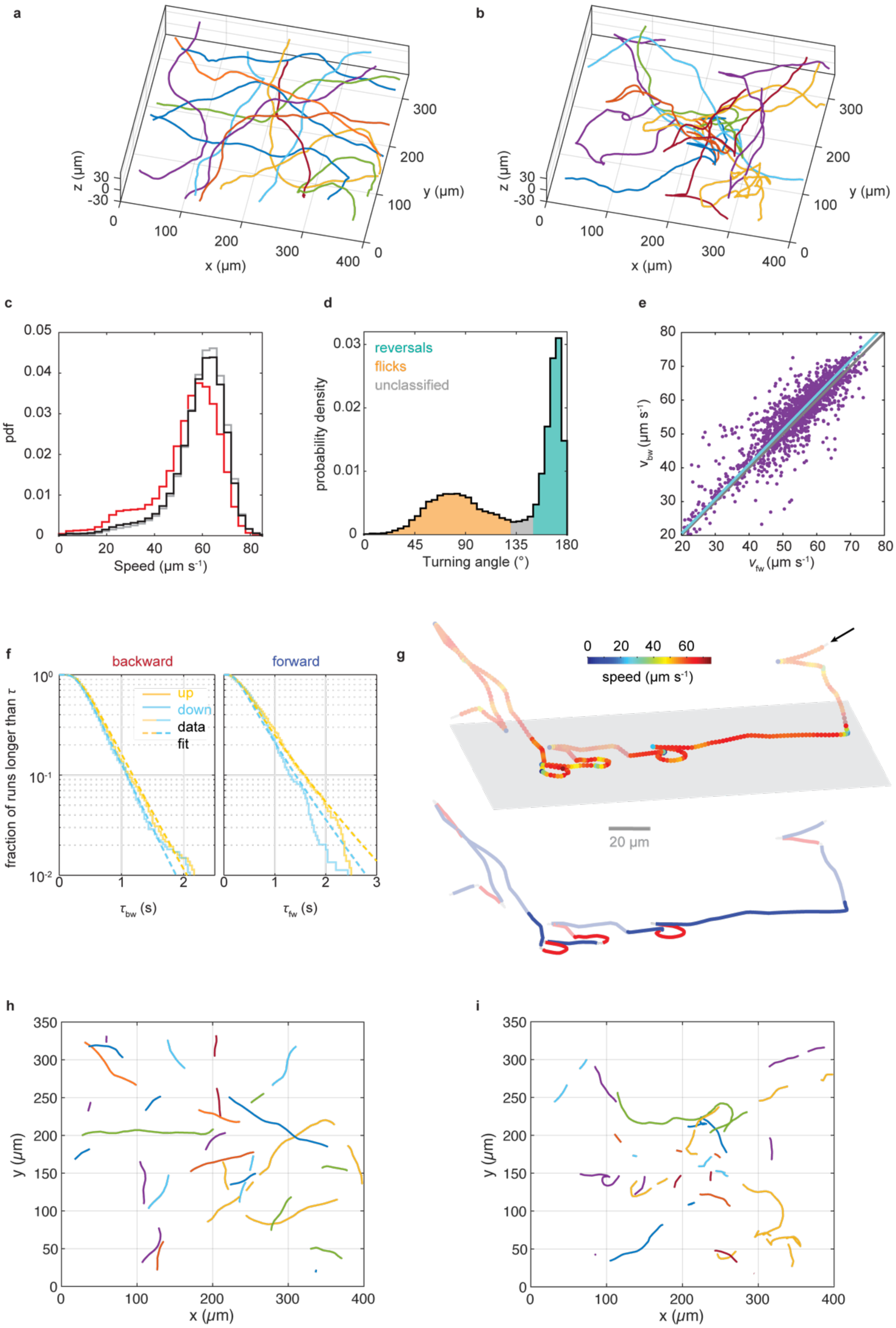

Supplementary Figure 2: Additional characterization of *C. crescentus* motility and chemotaxis. Examples of trajectories whose bulk segments show a) no turns and b) a turning frequency close to the population average. c) Instantaneous swimming speed distributions for the full motile bulk population (black, set 2 in Supplementary Table 2), the smooth-swimming population (grey, set 4) and that retained for run duration analysis (red, set 7), containing all trajectories with at least one run of defined duration and bacterial orientation. We attribute the subtle deviation in average swimming speed to selection bias, rather than biological differences. Conditions that are more likely to be met by longer trajectories select for bacteria of lower speed because they spend more time in the field of view. d) Distribution of turning angles and turn classification. Turns by an angle smaller than 130° are considered flicks and those by an angle larger than 150° are considered reversals. Flick angles have a magnitude of 80° ± 24° (mean ± standard deviation). e) Backward and forward swimming speed (v_{bw} and v_{fw}, respectively) in the same individuals reveal that backward runs are approximately 2.5% faster. Cyan: orthonormal linear fit with zero intercept (grey: unity, for comparison). f) Fraction of runs longer than τ for backward and forward runs, going up (yellow) or down (cyan) the gradient, defined as falling within a 36° cone around the positive or negative *x*-axis, respectively. The dashed lines show corresponding maximum-likelihood inverse Gaussian distribution functions (Methods). g) Example trajectory color-coded by speed (top) and run direction (bottom, red: backward, blue: forward), with near-surface segments shown at higher saturation than bulk segments. Arrow marks trajectory start. h,i) Surface segments (within approximately 4 µm of the top and bottom surface) of the example trajectories shown in a and b, with no turns (h) or a near-average turning frequency (i).

Supplementary Figure 3

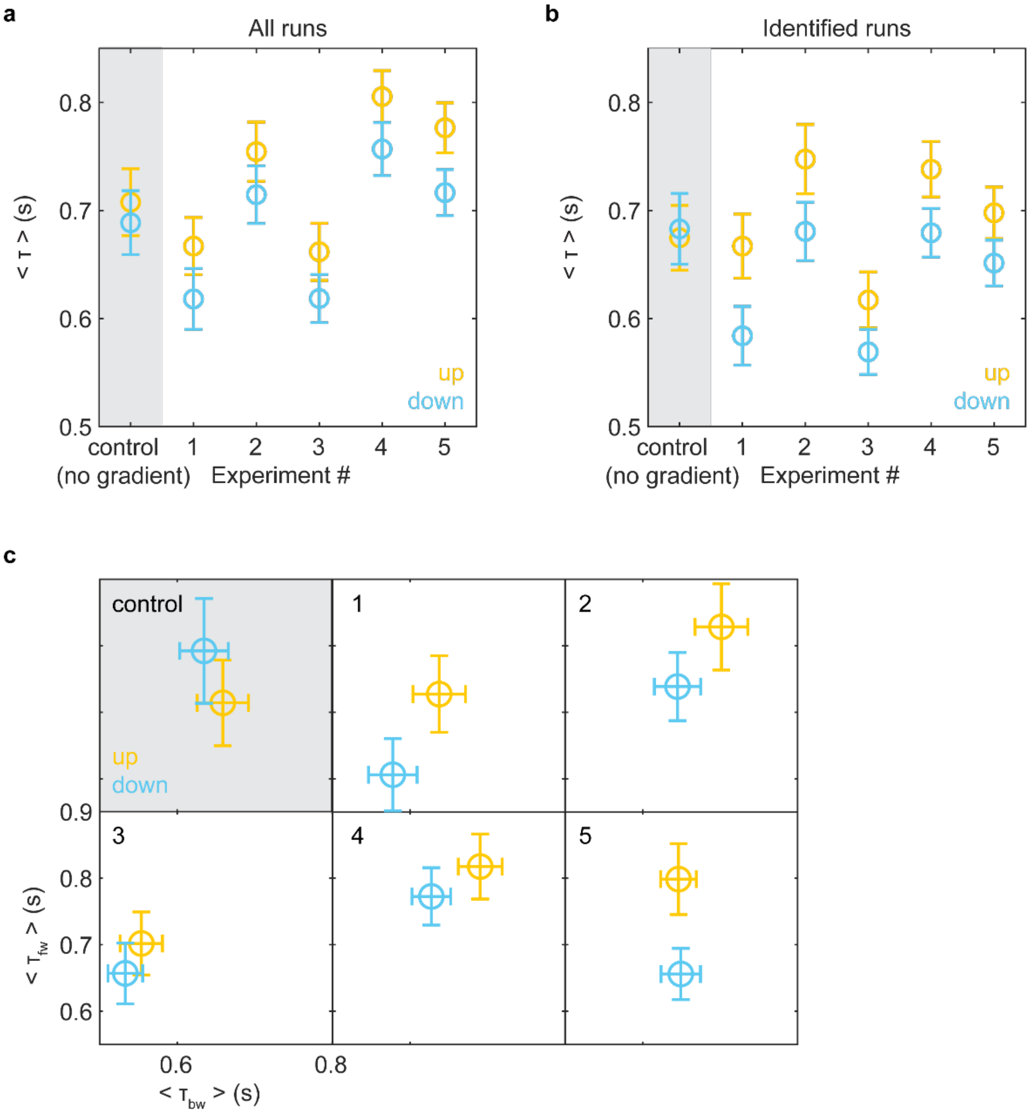

Supplementary Figure 3: Reproducibility of *C. crescentus* chemotaxis experiments. a,b) Average run durations of runs leading up or down the gradient (defined as in Figure 2) in 5 biological replicates in the presence of a 1 mM mm^{-1} xylose gradient as well as one control experiment without a gradient, a) for all runs, b) for runs of identified orientation (backwards/forwards). c) Average durations of forward versus backward runs in each of the experiments in panels a and b, for runs leading up (yellow) or down (cyan) the gradient. Error bars represent standard errors of the means.

Supplementary Figure 4

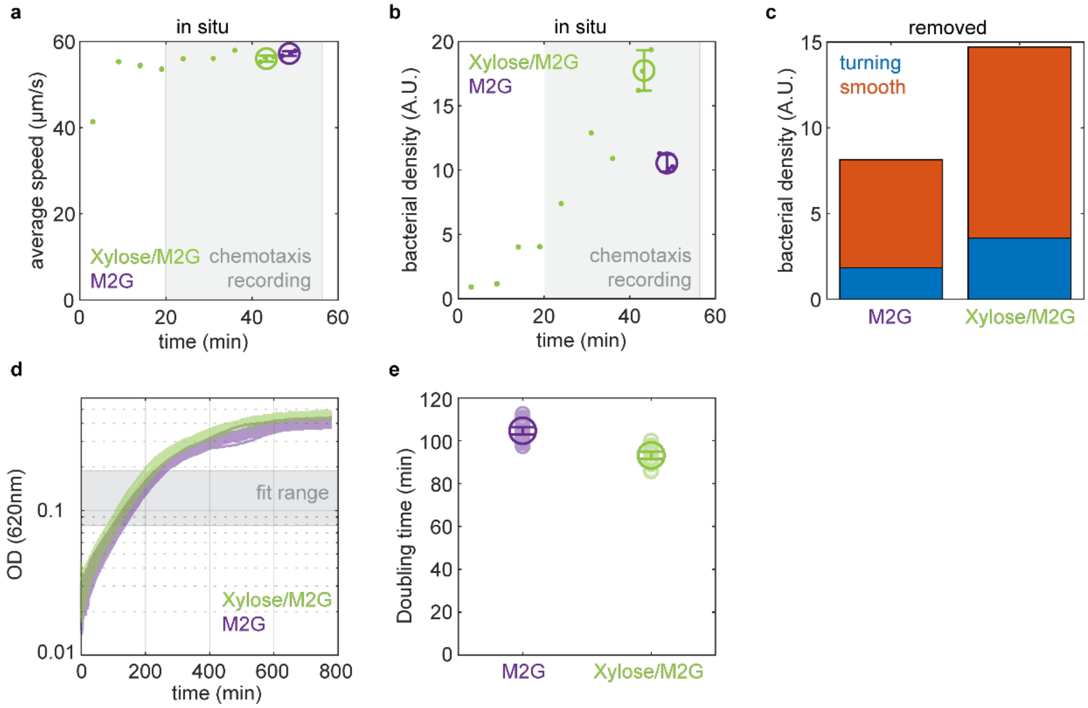

Supplementary Figure 4: Xylose-driven bacterial density imbalance. a) Average swimming speeds observed over time in the left and right reservoirs of the chemotaxis chamber, containing xylose/M2G and M2G, respectively. The grey box marks the time period during which trajectories are recorded in the gradient in the middle of the chamber during chemotaxis experiments. b) Bacterial densities observed in the reservoirs over time. In panels a and b, small points indicate individual 50-s recordings, and circles with error bars show the mean and standard deviation of three such recordings obtained in close temporal proximity. c) Bacterial density of solutions retrieved from the two reservoirs after 53 min. d) Individual optical density at 620 nm from microplate reader measurements in 96-well plate, after blank subtraction, for 20 wells with M2G or 20 wells with xylose/M2G. e) Doubling times, determined by a linear fit to the logarithm of the optical density data in panel d between OD 0.08 and OD 0.18 against time, indicate that the growth rate is approximately 11% higher in the presence of xylose. Error bars reflect 95% confidence intervals of average doubling times across 20 wells for each condition.

Supplementary Figure 5

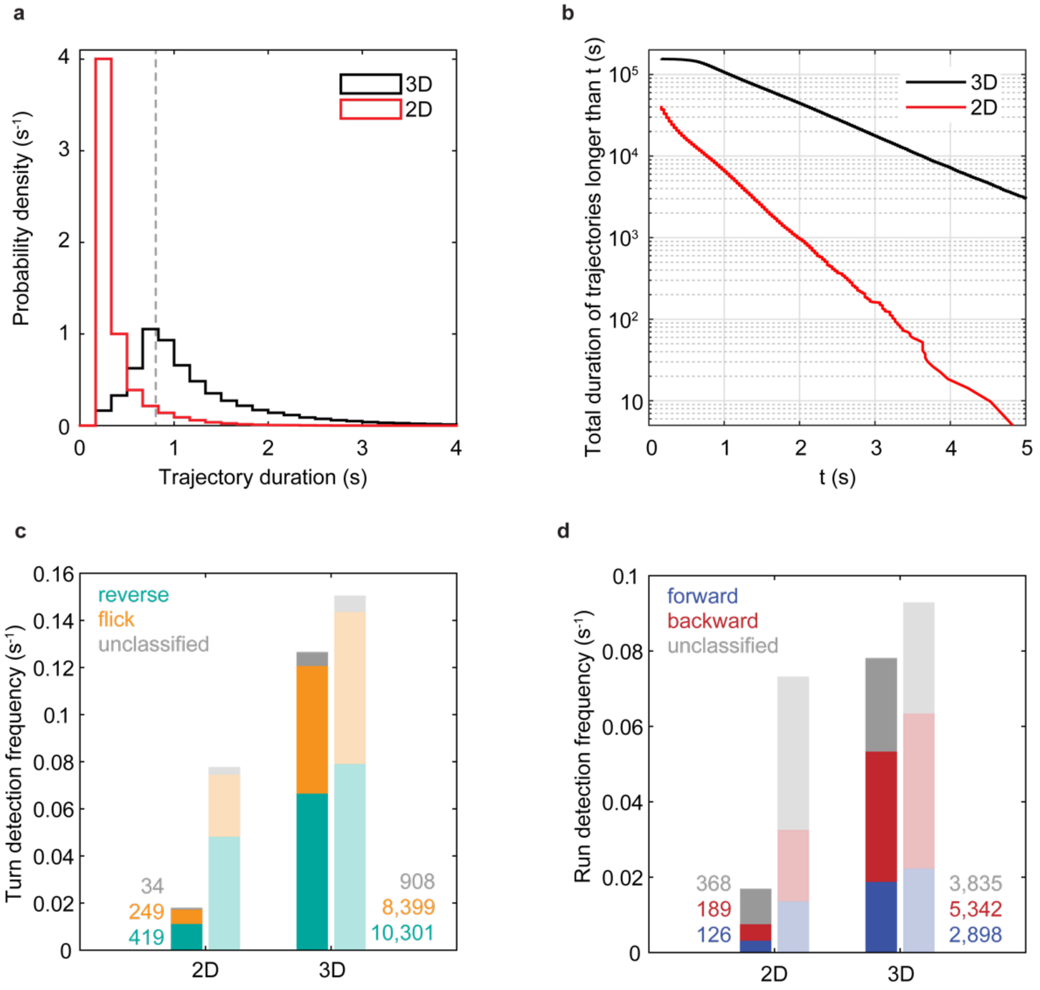

Supplementary Figure 5: 3D versus 2D tracking of *C. crescentus*. a) Probability distribution function of trajectory durations for full 3D motile bulk data (black, subset 2 in Supplementary Table 2) and the same cropped to an 8-µm thick central slice, corresponding to a typical depth of field used in 2D tracking (red, see Supplementary Discussion for details). Trajectories shorter than 5 frames were not retained for analysis. The grey dashed line indicates the minimum trajectory duration threshold used for turn detection in our study. b) Cumulative duration of trajectories whose duration exceeds a given threshold for both data sets. The exponential decay time scales of the distributions' tails are 2.0 s and 0.88 s for the 3D and the 2D case, respectively. c) Frequency of detected turning events for both data sets, normalized either across the full trajectory data set (left bars, corresponding to subset 2 for the 3D case) or that of trajectories that meet the minimum duration threshold of 0.8 s (right bars, faded, corresponding to subset 3 for the 3D case). We include only turns that allow a turning angle to be measured. Numbers in panels c and d indicate absolute numbers of events detected. d) Frequency of detected run types for the same normalizations and data sets as in panel c. 2D microscopy identifies the direction for far fewer run events than 3D microscopy because it undersamples flick events which typically result in the trajectory leaving the focus.

Supplementary Discussion

1. Consistency of measured *E. coli* drift speed with literature values

Very few methods allow direct measurements of the drift velocity. Colin et al.[1] used a highly sensitive population-scale approach to determine a drift velocity, v_d, normalized by the product of the motile fraction, α, and average speed, v_m, for AW405 of $v_d/(\alpha v_m) \sim 0.08$-$0.09$ for a 10 μM mm^{-1} MeAsp gradient, which translates in our case ($\alpha = 1$, $v_m = 29.9$ μm/s) to an expected drift of 2.4 - 2.7 μm s^{-1}, in excellent agreement with our result of 2.7 ± 0.3 μm s^{-1}. Recently, Schauer et al.[2] presented drift velocities directly computed from 2D trajectory data for the much slower swimming strain MG1655 (mean speed 16 μm s^{-1}). In addition to the strain difference, these results cannot be compared quantitatively to ours because the 2D approach introduces biases that are hard to correct[2]. For example, mandating a minimum trajectory duration in 2D limits the analyzed set of bacteria to those that move within the focal plane, which results in an over-estimate of the drift velocity as it discriminates against bacteria that move perpendicular to the gradient.

Other publications refer to the chemotactic sensitivity. Ahmed et al.[3] infer the drift velocity from the population-averaged swimming speed and the measured asymmetry in the time spent swimming up versus down the gradient for various gradient conditions, and then use the following relation to determine the chemotactic sensitivity:

$$\chi_0 = \frac{8v_{2\mathrm{D}}\left(K_\mathrm{d} + C(x)\right)^2}{\pi K_\mathrm{d}} \operatorname{arctanh}\left(\frac{3\pi}{8v_{2\mathrm{D}}} V_\mathrm{drift}\right) \frac{\mathrm{d}x}{\mathrm{d}C}$$

where $v_{2\mathrm{D}} = \frac{\pi}{4} v_{3\mathrm{D}}$ is the average bacterial swimming speed, K_d is the receptor/ligand dissociation constant, V_{drift} is the chemotactic drift, C is the MeAsp concentration and x the position in the gradient. Using this relation with $\mathrm{d}C/\mathrm{d}x = 1 \times 10^{-5}$ mM μm^{-1}, $C(x) = 5$ μM, and $K_d = 0.125$ mM[4], we obtain $\chi_0 = 11 \pm 1 \times 10^{-4}$ cm^2 s^{-1} from our drift velocity value of 2.7 ± 0.3 μm s^{-1}, which aligns well with previously reported values for the same strain (2.4×10^{-4} $cm^2 s^{-1}$ [5], 5×10^{-4} cm^2 s^{-1} [6], 12.4×10^{-4} cm^2 s^{-1} [3]).

2. *C. crescentus* chemotaxis

2.1 Nature of the smooth-swimming subpopulation.

While, even for a uniform population of turning bacteria, it is expected that a subset of trajectories exhibits no turning events by random chance, several observations support the notion that the trajectories without turns contain a phenotypically distinct subpopulation: Firstly, they show a vastly different drift velocity from the turning population (Supplementary Table 2, $p \approx 10^{-5}$ under a one-sided two-sample t-test on drift velocity averages obtained for sets of 150 trajectories each). Secondly, the observed fraction of trajectories with no turns exceeds the fraction expected from the average turning frequency by 9%, assuming Poisson statistics and a uniform turning frequency across the population. An average turning frequency of 0.34 Hz is obtained by dividing the number of turns observed across the entire turn detection dataset by

the set's total trajectory time. Thirdly, if we determine the turning frequency as the value that best predicts the observed relative frequency of trajectories with 1, 2, and 3 turns under the assumption of Poisson statistics, ignoring the frequency of trajectories with no turns, we obtain a higher rate of 0.36 Hz. Based on this value, the number of trajectories with zero turns is 24% higher than expected.

Although turning events are not truly Poissonian, as indicated by the peaked, non-exponential distribution of run durations, we do not anticipate that this changes the conclusion that smooth trajectories are overrepresented because the minimum trajectory duration of 0.8 s we impose is located on the exponential tail of the run duration probability distribution function. In fact, the Inverse Gaussian distributions we extract predict a larger number of runs shorter than 0.8 s than an exponential distribution with the same mean, so we are likely underestimating the overrepresentation of smooth trajectories.

It is possible that our population contains a continuous spectrum of turning rates rather than two discrete, turning and non-turning, subpopulations. The run duration distribution of the turning population, however, is well-captured by a single distribution (Fig. 2b).

Multiple possible causes could potentially underlie the smooth-swimming subpopulation we observe. One possibility is that these cells are experiencing the receptor degradation that accompanies the swarmer-to-sessile cell transition[7]. In *E. coli*, the tumble signal [CheY-P] is produced by the receptor-bound kinase CheA, so that a deletion of chemoreceptors, like a deletion in *cheA* or *cheY*, produces a smooth-swimming phenotype[8,9]. In *C. crescentus*, however, a deletion in *cheAI* from the major chemotaxis operon has been reported to result in an increased turning frequency[10], thus it is unclear whether a receptor deletion or degradation would yield a smooth-swimming phenotype in this species.

A second possible explanation might lie in the c-di-GMP-activated Cle proteins which were recently shown to induce smooth-swimming, likely by competing with CheYII, thought to be the main chemotaxis response regulator, for binding sites at the motor[11].

2.2 Motor bias of the smooth-swimming subpopulation.

In principle, it is possible for smooth swimmers to exhibit the same motor bias as turning cells. The bias would manifest itself in the fraction of smooth swimmers that swim in pushing vs pulling mode.

Trajectory curvature at the surface can, in principle, enable a determination of swimmer orientation. High curvature segments typical of the pulling mode are readily apparent in surface segments of example trajectories from the turning population, but not from the smooth-swimming population (Supplementary Fig. 2h,i). Forward swimming thus seems more likely for the smooth-swimming population, but we cannot exclude backward swimming because not all trajectories contain surface segments and interactions with stalked bacteria at the surface may disrupt surface trajectory curvature.

Assuming that there are two discrete underlying phenotypes (smooth-swimming and turning), the set of trajectories that do not contain turning events would be expected to contain both true smooth swimmers as well as individuals from the turning population which only happen to not turn during the recorded trajectory segment. Thus, if high curvature surface segments were observed in trajectories without turns, it would not rule the possibility that smooth swimmers only swim forward.

Finally, it is, however, also plausible that the biological factors that distinguish the smooth-swimming and turning phenotypes also alter the motor bias. We cannot distinguish these possibilities on the basis of our data.

2.3 Diffusive drift due to imbalance in bacterial densities

A bacterial concentration gradient drives a diffusive bacterial drift of

$$v_{\text{diff}} = \frac{J}{b} = -\,\mathrm{D}\frac{\frac{\mathrm{d}b}{\mathrm{d}x}}{b(x)} = -\mathrm{D}\,r(x)\,,$$

where $b(x)$ is the position-dependent bacterial density, D is an effective diffusion coefficient, and $J(x)$ is the resulting diffusive flux, and $r(x)$ is the relative bacterial density gradient. In our device, we expect a linear gradient across a d = 1 mm long channel between reservoirs with concentrations $b_0 = c\,b_1$ and b_1, where $c < 1$. The relative gradient in the center of the device is then

$$r(d/2) = \frac{2(1-c)}{d(1+c)}$$

and the resulting diffusive drift at this position is

$$v_{\text{diff}}(d/2) = -\frac{2D(1-c)}{d(1+c)}.$$

The effective 3D diffusion coefficient for run-reverse-flick motility with constant speed v and two different run durations was derived by Taktikos et al.[12], see (Eq. 24):

$$D = \frac{v^2}{3}\,\frac{\tau_{\text{bw}}^2 - \tau_{\text{bw}}\tau_{\text{fw}}(1-2D_{\text{r}}\tau_{\text{bw}}) + \tau_{fw}^2(1+2D_{\text{r}}\tau_{\text{bw}})}{(\tau_{\text{bw}}+\tau_{\text{fw}})(1+2D_r\tau_{\text{bw}})(1+2D_{\text{r}}\tau_{\text{fw}})}$$

The effective rotational diffusion coefficient D_r is likely very small for *C. crescentus*. Assuming τ_{fw} = 0.75 s and τ_{bw} = 0.64s, v = 56 µm/s, and D_r = 0, we obtain D = 370 $\mu m^2\,s^{-1}$. If $D_r = 0.1\ s^{-1}$, which is likely an overestimate, we obtain D = 363 $\mu m^2\,s^{-1}$.

For the apparently non-turning population, which shows negative drift, we do not know with certainty whether the bacteria do not turn at all or still perform run-reverse-flick motility with much longer run durations. Assuming $\tau_{fw} = \tau_{bw}$ = 2 s, v = 59 µm/s and $D_r = 0.1\ s^{-1}$, we obtain D = 1067 $\mu m^2\,s^{-1}$.

We note that these diffusion coefficient estimates are coarse as they rely on simplifying assumptions. For example, we use population-averaged values for input parameters and do not take into account variability between individuals, which may lead to inaccuracies in the diffusion coefficient due to its nonlinear dependence on input parameters. The run durations used may be underestimates as long runs tend to be under-sampled[13], thus the diffusion coefficients are likely underestimates. The diffusion coefficient estimates also do not take into account the complex geometry of the assay. For processes with short run length, the behavior may be well approximated as 3D, but swimmers with longer run lengths will frequently interact with the walls. Such interactions could both increase the effective diffusion coefficient via dimensional reduction and decrease it via surface interactions such as circling.

Bacterial density measurements for the two reservoirs in the chemotaxis device indicate that the xylose-containing reservoir has an approximately 1.8-fold higher density than the one that only contains buffer, corresponding to c = 1/1.8 = 0.56. Based on our diffusion coefficient estimates above, we thus expect a diffusive drift of approximately -0.2 $\mu m\,s^{-1}$ for the turning population

and -0.6 µm s^{-1} for the smooth-swimming population. The latter value is in good agreement with the experimentally determined values of –(0.8 ± 0.2) µm s^{-1} for smooth swimmers, indicating that the experimentally observed drift of smooth swimmers may be entirely due to effective diffusion, with no chemotactic component. The value of -0.2 µm s^{-1} estimated for the turning population agrees well with the difference, –(0.17 ± 0.33) µm s^{-1}, between their measured drift velocity of (0.26 ± 0.12) µm s^{-1} and their chemotactic drift expected on the basis of their run duration modulation, (0.43 ± 0.21) µm s^{-1}. We thus conclude that the drift velocity observed for the turning population likely slightly underestimates the chemotactic drift velocity due to a diffusive counter flux.

2.4 Density imbalances driven by a chemoattractant-conferred growth benefit

Many chemoattractants are nutrients and confer a growth benefit. Let the growth rates in the presence and absence of chemoattractant be μ_1 and μ_2, respectively, with $\mu_1 > \mu_2$. If bacterial suspensions with and without chemoattractant, but with identical initial density N_0 are filled into the reservoirs at $t = 0$, their densities N_i will evolve as $N_i(t) = N_0\, e^{\mu_i t}$, and the ratio of their densities as $R_\mathrm{s}(t) = N_1/N_2 = e^{(\mu_1-\mu_2)t}$. Under the conditions of the experiment, that is, room temperature and a minimal medium that does not support growth well, the growth rate is typically very low and the duration to the experiment substantially shorter than the duplication time, $t_\mathrm{d} = \ln(2)/\mu$, $t < t_\mathrm{d}$, so that $R_\mathrm{s}(t) \approx 1 + (\mu_1 - \mu_2)t$. Small growth rate differences thus result in only small density differences between reservoirs over the course of a typical suspension-based experiment.

In our *C. crescentus* assay, however, the observed bacteria are initially generated only by growth from a reservoir of stalked bacteria at the surface with density N_r, at a constant rate. At longer times, exponential growth from the generated swarmer cells is expected to drive the dynamics: $\frac{dN_i}{dt} = \mu_i N_r + \mu_i\, N_i(t)$, thus $N_i(t) = N_r(e^{\mu_i t} - 1)$. For simplicity, we assume the same growth rate μ_i for growth from the surface reservoir and growth from new swarmer cells. We also ignore any possible delays due to cell cycle timing in the transition of swarmer cells back to stalked cells that is required to enable cell division. The density ratio then evolves as $R_\mathrm{r}(t) = \frac{e^{\mu_1 t}-1}{e^{\mu_2 t}-1}$. Hence even the initial ratio already reflects the growth rate difference, $R_\mathrm{r}(t) \approx \mu_1/\mu_2$ for $t \ll t_\mathrm{d}$, while in the suspension assay, $R_\mathrm{s}(0) = 1$. At long times, exponential growth from the generated swarmer cells is expected to drive the dynamics. This will occur when their population becomes sufficiently large that their contribution to growth becomes comparable to or larger than that provided by the initial stalked cell carpet. Eventually, the ratio density will grow with the same scaling as for growth from a suspended population, $R_\mathrm{r}(t) \propto e^{(\mu_1-\mu_2)t}$. The transition from the linear to the exponential regime is expected to occur around the doubling time, t_d. Most importantly, the density ratio is larger for the reservoir-driven assay than for the suspension assay, $R_\mathrm{r}(t) > R_\mathrm{s}(t)$, at all times.

Our experimentally observed density ratio of $R_\mathrm{r} \approx 1.8$ is substantially larger than the growth rate ratio, $\mu_1/\mu_2 \approx 1.1$, inferred from plate reader experiments. Thus, either the growth dynamics in our chambers have already left the linear growth regime, or the growth conditions in the plate reader do not reflect the growth in the chemotaxis chambers well. Both possibilities are plausible, and the latter very likely, as the plate reader experiments were performed at a higher temperature.

Thus, we conclude that, in typical assays where a suspension of bacteria is injected into the chemotaxis device, any density imbalances incurred by a growth benefit conferred by the chemoattractant will be typically be much smaller than in the *C. crescentus* assay presented here.

2.5 Motor switching model.

The ratio of probabilities, p, of being in the CW vs the CCW state is determined by their energy difference ΔE_b (Figure 2d, e for definitions of terms) between them: $p(\text{CW})/p(\text{CCW}) = \exp(-\Delta E_b/k_B T)$, where k_B is the Boltzmann factor and T is the absolute temperature. The motor's CW bias is given by p(CW). The rates of switching between states depend on the energy barrier separating the states: $k_{CCW} \propto \exp(-\Delta E_{CW}/k_B T)$, $k_{CW} \propto \exp(-\Delta E_{CCW}/k_B T)$.

In the *E. coli* model, CheY-P binding is thought to destabilize the CCW state while stabilizing the CW state so that ΔE_b decreases (thus increasing the motor's CW bias), ΔE_{CCW} decreases (increasing k_{CW}), and ΔE_{CW} increases (decreasing k_{CCW})[14].

For an alternative model that enables chemotaxis at constant motor bias, ΔE_b must remain constant. CheY-P binding can then modify switching rates by either destabilizing both motor rotation states by the same amount[15] or by lowering the energy barrier between them.

While our data are consistent with the notion that the motor bias remains constant during chemotaxis, we cannot exclude that experimental scenarios that evoke a stronger chemotactic response might exhibit a deviation from a constant bias. We emphasize, however, that such a deviation would only entail minor modifications to the model proposed here and would not compromise the key point of our findings, namely that chemotaxis is achieved by extending either motor rotation interval if favorable, as opposed to modifying their relative durations.

3. Data requirements for resolving chemotactic drift from individual trajectories

Regardless of the method used, the amount of data available sets a limit on the precision with which we can determine averages as per the central limit theorem. Suppose one acquired a large number, N, of independent samples of velocity vectors from a population of bacteria swimming in random directions in 3D with fixed absolute speed, v_0. In the presence of chemotaxis, the expected drift velocity in the gradient direction is v_d. The measured drift velocity is $v_m = \langle v_x \rangle$ where v_x is the component of the swimming velocity along the gradient direction. In the absence of chemotaxis, v_x has a flat distribution on the interval $[-v_0, v_0]$ with a standard deviation of $v_0/\sqrt{3}$. According to the central limit theorem, the standard error of v_m is then $SE(v_m) = v_0/(\sqrt{3N})$. If the drift velocity is small compared to the swimming speed, $v_d \ll v_0$, we can approximate the effect of chemotaxis as a shift of the entire distribution in v_x by v_d. In this approximation, the distribution's mean shifts from 0 to v_d, but the standard deviation remains unchanged. The confidence in a given measured drift, v_m, is determined by the ratio $f = SE(v_m)/|v_m| = \frac{v_0}{\sqrt{3N}\,|v_m|}$. E.g., a 98% confidence that the measured drift v_m represents a true underlying v_d larger than zero requires $f < 0.5$. Conversely, the number of independent data points required to achieve a ratio f follows $N_{\min} = \frac{1}{3f^2}\left(\frac{v_0}{|v_m|}\right)^2$.

Experimentally, each trajectory can be considered an independent measurement. In principle, trajectories longer than the autocorrelation time of the velocity orientation vector can yield multiple independent velocity measurements. For *C. crescentus*, velocity orientations are highly correlated over the course of a forward run and the next backward run which retraces it, but the subsequent flick effectively randomizes the orientation. The autocorrelation time scale, τ_c, can thus be estimated as the sum of the average durations of forward and backward runs, $\tau_c \approx \langle\tau_{fw}\rangle + \langle\tau_{bw}\rangle \approx 0.79$ s + 0.66 s = 1.45 s, where we have used the average run durations obtained for runs leading up the gradient. This value is very similar to the average trajectory duration of 1.55 s for the dataset; thus, we ignore this subtlety and base our estimates on the number of trajectories.

For *E. coli*, a typical experiment comprises $N \approx 3 \times 10^3$ trajectories. With $v_m = 2.7$ µm s^{-1} and $v_0 =$ 30 µm s^{-1}, we estimate $f = 0.12$, consistent with the approximately 10% variation in results between experiments. With our values for *C. crescentus*, $v_0 = 56$ µm s^{-1}, $v_m = 0.26$ µm s^{-1}, and $N \approx 7.9 \times 10^4$, we expect $f = 0.43$ based on solely statistical errors, close to the $f = 0.46$ estimated experimentally.

The data requirements for detecting the drift velocity of *C. crescentus* and *E. coli* in our essay are thus accounted for by statistical considerations.

4. Comparison of 3D and 2D tracking

To compare the performance of 3D tracking to a common 2D tracking approach, we construct a 2D comparison dataset from our *C. crescentus* 3D data based on an estimate of the constraints of 2D tracking. 2D tracking studies often observe bacteria in a slice whose vertical thicknesses is determined by the depth of field of the objective lens used. This value can range from about 2 µm for a 40x, NA 0.60, lens like the one used here to about 8 µm for a 10x, NA 0.3, lens, assuming a camera pixel size of 6 µm. To construct a realistic 2D data set, we crop our 3D *Caulobacter crescentus* bulk trajectories, which span approximately 44 µm in *z*, to those segments contained in a slice of a vertical height of 8 µm, positioned at the center of our chamber in *z*, and discard the *z* information for the resulting data set.

Not only does the 2D dataset comprise less data by overall trajectory duration because it covers a smaller volume, but it also consists of much shorter trajectories (Supplementary Fig. 5a) because trajectories end when bacteria leave the slice. Many behavioral parameters only become detectable in trajectories with a certain minimum trajectory duration. For example, unambiguous identification of bacterial orientation during a run requires that both turning events before and after the run can be identified as reversals or flicks. The amount of trajectory data that meets a given duration threshold decreases exponentially with the threshold value. For the 2D data set, the rate of decrease is more than twice as fast as for the 3D data set (Supplementary Fig. 5b). We select a minimum duration threshold of 0.8 s, slightly longer than the average run duration, to limit analysis to trajectories that are likely to capture runs of detectable orientation. At this threshold value, the amount of data in the 2D data set is reduced by more than an order of magnitude compared to the 3D data set.

Next, we compare the frequencies of the different types of turning events detected in 2D and 3D tracking. To detect turning events in the 2D data, we follow the same procedure as in 3D, except

speeds and rates of angular change were only computed from the x and y components of the position data and the detection threshold factor was adjusted to a value of 14 to minimize the difference in the number of detected events compared to a full 3D treatment of the same data. 2D tracking underestimates the number of flicks because flicks often result in the bacterium leaving the observation volume. By contrast, reversals result in the bacterium backtracking along its previous path, so a forward run in the observation volume is typically followed by a backward run that is also in the observation volume. As a result, we detect nearly twice as many reversals as flicks in the 2D data set, compared to a nearly balanced ratio of 1.2 in the 3D data set (Supplementary Fig. 5c).

The undersampling of flicks in 2D tracking limits the ability to assign bacterial orientations to runs, as we require a flick to be detected at one end and a reversal to be detected at the other end of a run to unambiguously distinguish forward and backward swimming. As a result, a bacterial orientation can only be assigned to less than half of the runs observed in 2D tracking, compared to nearly 70% for 3D tracking (Supplementary Fig. 5d).

Because forward runs are, on average, longer than backward runs, examining forward and backward runs separately is crucial to being able to detect whether run durations depend on orientation relative to the gradient. The 2D dataset detects only 210 runs of known bacterial orientation, of which fewer than 80 are aligned up or down the gradient. As a result of the low rate of detection of runs with known bacterial orientation, the 2D data set is unable to distinguish run durations up and down the gradient. To detect a similar number of runs with known bacterial orientation as in 3D tracking, approximately 30-40 times as much data would need to be recorded, corresponding to 1.5-2 full days of recording, compared to the 75 min on which the 3D dataset is based.

Supplementary Table 1

Dataset		Figures		# of motile trajectories	total motile trajectory duration (s)	motility threshold (µm s^{-1})	average motile speed (µm s^{-1})	v_d (µm s^{-1})
Speed in reservoirs over time (min)	3	S. Fig. 1d		118	810	10	21.0	N/A
	7			118	908	10	20.2	N/A
	10			150	1,155	10	20.6	N/A
	50			164	1,243	10	19.8	N/A
Geometry tests MeAsp	-1 mM mm^{-1}	S. Fig. 1e		3,589	13,825	10	20.4	5.0
	1 mM mm^{-1}			6,497	19,926	10	20.7	5.2
	no gradient			1,996	7,470	10	20.8	0.3
10 µM mm^{-1} MeAsp	same day triplicates (all)	Fig. 1d, S. Fig. 1g	Fig.1b, S. Fig. 1f	5,061	36,979	15	30.4	1.2
				2,498	20,879	15	30.4	1.5
				2,344	21,704	15	29.6	1.0
	same day triplicates (bulk)	Fig. 1e		4,488	12,284	15	30.3	2.5
				2,541	7,153	15	30.5	3.1
				2,265	6,892	15	29.4	2.6
	additional replicates (bulk)			11,490	29,947	15	32.4	2.5
				10,277	29,003	15	30.1	2.7

Supplementary Table 1: Statistical descriptors of *E. coli* datasets and their corresponding figures. Bulk data, meaning containing only trajectory segments that are at least 10 µm from surfaces, are presented, unless otherwise specified.

Supplementary Table 2

set #	set	sub-set of	additional conditions re parent set	# of trajec-tories	total trajectory time (s)	mean trajectory duration (s)	mean speed (µm s^{-1})	drift velocity in x (µm s^{-1})	drift velocity in y (µm s^{-1})
**1**	all	1	-	125,208	249,729	2.0	50.4	-0.20 ±0.07	-0.09 ±0.07
**2**	motile bulk	1	>13 µm from surface, v_m > 20 µm s^{-1}	130,243	154,952	1.2	58.2	-0.22 ±0.09	-0.09 ±0.11
**3**	turn detection set	2	T > 0.8 s	79,244	123,001	1.6	57.7	-0.31±0.11	-0.09 ±0.11
**4**	smooth swimmers	3	0 turns	49,753	66,949	1.3	59.2	-0.80 ±0.17	-0.14 ±0.18
**5**	turning	3	1+ turns	29,491	56,054	1.9	55.8	0.26 ±0.12	-0.03 ±0.12
**6**	run analysis	5	1+ run[a]	8,409	21,848	2.6	52.5	0.26 ±0.18	0.16 ±0.18
**7**	forward/ backward analysis	6	1+ defined run[b]	6,230	16,368	2.6	53.6	0.22 ±0.19	0.21 ±0.22
**8**	forward/ backward speed analysis	7	1+ fw run AND 1+ bw run	1,418	4,896	3.5	50.8	0.01 ±0.35	0.19 ±0.29

Supplementary Table 2: Definitions and properties of subsets of *C. crescentus* trajectories used for analysis. The number of trajectories in subset 2 increases relative to subset 1 because the condition of a minimum distance to the surface segments trajectories into potentially multiple rejected or retained parts. v_m: mean swimming speed, *T*: trajectory duration. [a] A run is included here if its beginning and end are detected. [b] Defined runs are those whose bacterial orientation can be determined based on the magnitude of the preceding and subsequent turns. Error estimates are standard errors.

References

1 Colin, R., Zhang, R. & Wilson, L. G. Fast, high-throughput measurement of collective behaviour in a bacterial population. *J Roy Soc Interface* **11**, 20140486-20140486, doi:10.1098/rsif.2014.0486 (2014).

2 Schauer, O. *et al.* Motility and chemotaxis of bacteria-driven microswimmers fabricated using antigen 43-mediated biotin display. *Sci. Rep.* **8**, 9801, doi:10.1038/s41598-018-28102-9 (2018).

3 Ahmed, T. & Stocker, R. Experimental Verification of the Behavioral Foundation of Bacterial Transport Parameters Using Microfluidics. *Biophysical Journal* **95**, 4481-4493, doi:10.1529/biophysj.108.134510 (2008).

4 Mesibov, R., Ordal, G. W. & Adler, J. The Range of Attractant Concentrations for Bacterial Chemotaxis and the Threshold and Size of Response over This Range : Weber law and related phenomena. *The Journal of General Physiology* **62**, 203-223, doi:10.1085/jgp.62.2.203 (1973).

5 Lewus, P. & Ford, R. M. Quantification of random motility and chemotaxis bacterial transport coefficients using individual ‐ cell and population ‐ scale assays. *Biotechnol. Bioeng.* **75**, 292-304, doi:10.1002/bit.10021 (2001).

6 Ahmed, T., Shimizu, T. S. & Stocker, R. Bacterial Chemotaxis in Linear and Nonlinear Steady Microfluidic Gradients. *Nano Letters* **10**, 3379-3385, doi:10.1021/nl101204e (2010).

7 Alley, M. R., Maddock & Shapiro, L. Requirement of the carboxyl terminus of a bacterial chemoreceptor for its targeted proteolysis. *Science* **259**, 1754-1757, doi:10.1126/science.8456303 (1993).

8 Parkinson, J. S. cheA, cheB, and cheC genes of Escherichia coli and their role in chemotaxis. **126**, 758-770 (1976).

9 Wright, S., Walia, B., Parkinson, J. S. & Khan, S. Differential Activation of Escherichia coli Chemoreceptors by Blue-Light Stimuli. *J. Bacteriol.* **188**, 3962-3971, doi:10.1128/JB.00149-06 (2006).

10 Kovarik, M. L. *et al.* Microchannel-nanopore device for bacterial chemotaxis assays. *Anal. Chem.* **82**, 9357-9364, doi:10.1021/ac101977f (2010).

11 Nesper, J. *et al.* Cyclic di-GMP differentially tunes a bacterial flagellar motor through a novel class of CheY-like regulators. *eLife* **6**, 550, doi:10.7554/eLife.28842 (2017).

12 Taktikos, J., Stark, H. & Zaburdaev, V. How the Motility Pattern of Bacteria Affects Their Dispersal and Chemotaxis. *PLoS One* **8**, e81936-81916, doi:10.1371/journal.pone.0081936 (2013).

13 Taute, K. M., Gude, S., Tans, S. J. & Shimizu, T. S. High-throughput 3D tracking of bacteria on a standard phase contrast microscope. *Nature Communications* **6**, 1-9, doi:10.1038/ncomms9776 (2015).

14 Scharf, B. E., Fahrner, K. A., Turner, L. & Berg, H. C. Control of direction of flagellar rotation in bacterial chemotaxis. *Proc National Acad Sci* **95**, 201-206 (1998).

15 Cai, Q., Li, Z., Ouyang, Q., Luo, C. & Gordon, V. D. Singly Flagellated Pseudomonas aeruginosa Chemotaxes Efficiently by Unbiased Motor Regulation. *mBio* **7**, e00013, doi:10.1128/mBio.00013-16 (2016).
