# Supplementary material for: A multiscale 3D chemotaxis assay reveals bacterial navigation mechanisms
Source: Commun Biol. 2021 Jun 3;4:669. doi: 10.1038/s42003-021-02190-2 (PMC8175578; doi:10.1038/s42003-021-02190-2)
Supplement: Supplementary file 2 — Description of Additional Supplementary Files [file 42003_2021_2190_MOESM2_ESM.pdf]

Description of Additional Supplementary Files

File name: Supplementary Data 1

Description: All data shown in the main and Supplementary Figures is provided in Excel format as Supplementary Data 1. Each sheet corresponds to one figure.
